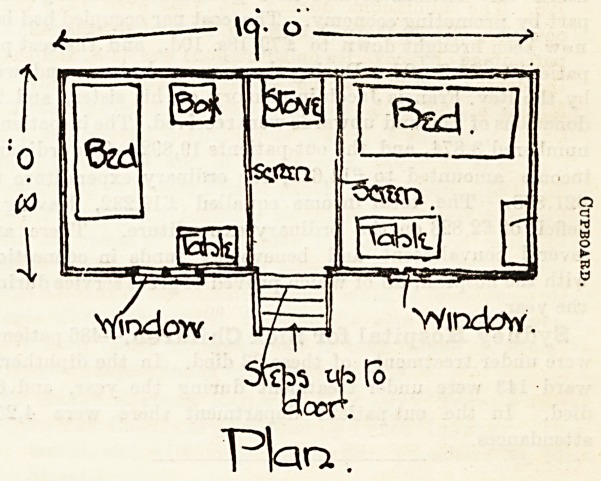# Practical Departments

**Published:** 1895-07-20

**Authors:** 


### PRACTICAL DEPARTMENTS.

### ISOLATION HOSPITALS FOR SMALL DISTRICTS.

The efficient treatment of infectious disease in country districts is often a matter of some considerable difficulty, yet it is quite certain that the prompt isolation of such cases is a matter of which the importance cannot be overrated. Too often it happens that an outbreak of scarlet fever or small pox gains disastrous hold upon a district, when the existence of means of isolation close at hand would have meant the saving not only of life but of much expense in the end.

The erection of a permanent hospital for these cases is not always possible or desirable in distant parts of country neighbourhoods, and it is when patients would consequently have to be conveyed some miles to the nearest institution that such an expedient as the "Hospital Caravan," of which we here give a sketch and plan, will be likely to be found of immense value.

It is the Local Authority of the Eastern District of Haddingtonshire, N.B., who have caused a caravan on these lines to be constructed by Messrs. H. and A. Brown, Springfield Street, Leith, for use in the district under their charge, for the isolation of infectious cases. This district comprises some eight parishes, containing a population of 5,817 persons, who are otherwise unprovided with hospital accommodation. It will be readily seen that in outlying districts of this description, with a widely scattered population, a hospital on wheels, which could travel about as required, might be quite the most efficient way to check any outbreak of infectious disease, besides being the most economical from the point of view of the ratepayers. Thus the conveyance of typhoid or diphtheria patients over a long distance might be entirely obviated, and in the case of an extended spread of an epidemic in one particular place the hospital accommodation could be added to *ad libitum*, provided a sufficient supply of these ambulances be kept at the head-quarters of the district, or given an arrangement of lending and borrowing with neighbouring localities adopting the same system.

Our sketch gives a rough idea of the caravan which is to do duty in the Haddington district. It has an air space of 1,520 cubic feet, it is 19 feet long by 10 feet high (from floor to roof), and 8 feet wide. It is on four wheels and can be drawn by two horses. The walls are double, with an intervening space of an inch and a half, and as they are easily removable a very thorough disinfection can be carried out. In the event of more than one such caravan being required (each being fitted with two beds) one end can be taken away to allow of the two being placed together, corridor-wise. It is supplied with five ventilators near the roof that shut and open by cords and pulleys on the same principle as Sherringham valves. There are also two Tobin tubes at each side, and centre ventilation in the ceiling in the shape of a shaft with double tube to carry off the warm air from the centre lamp. It is well provided with everything necessary, from the two spring beds to cooking utensils, and when required can be heated with a stove which is placed opposite the entrance and between the two beds, which are separated by screens or curtains. A tent is to be supplied with each caravan for the nurse or for cooking, thus making the hospital very complete; and the cost of each caravan, fully equipped, is estimated at £120.

The expense of keeping up anything like a staff is practically done away with, as the local doctor would be placed in charge of the hospital, and the nurse or nurses might be permanent or not according to the special circumstances, and as each district committee found most desirable. The arrangements as to the practical working of the ambulance would naturally vary according to local resources.

The plan thus started by the Haddington authorities seems to have much to recommend it for use in similar country districts, and we should be glad to see the example followed, and to hear with what success the scheme is attended in practice wherever attempted.

---

## Figures and Tables

**Figure f1:**
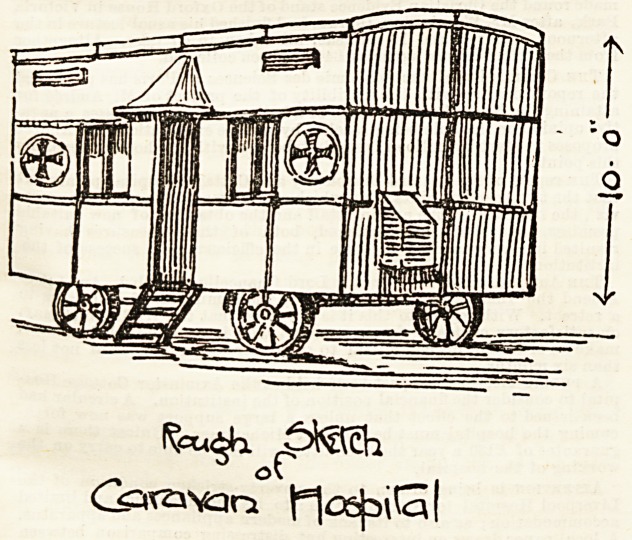


**Figure f2:**